# Role of non-coding RNAs in radiosensitivity of colorectal cancer: A narrative review

**DOI:** 10.3389/fonc.2022.889658

**Published:** 2022-07-22

**Authors:** Chun-Ming Huang, Hsiang-Lin Tsai, Yen-Cheng Chen, Ching-Wen Huang, Ching-Chun Li, Wei-Chih Su, Tsung-Kun Chang, Yung-Sung Yeh, Po-Jung Chen, Ming-Yii Huang, Jaw-Yuan Wang

**Affiliations:** ^1^ Department of Radiation Oncology, Kaohsiung Medical University Hospital, Kaohsiung, Taiwan; ^2^ Department of Radiation Oncology, Faculty of Medicine, College of Medicine, Kaohsiung Medical University, Kaohsiung, Taiwan; ^3^ Department of Radiation Oncology, Kaohsiung Municipal Ta-Tung Hospital, Kaohsiung Medical University, Kaohsiung, Taiwan; ^4^ Graduate Institute of Medicine, College of Medicine, Kaohsiung Medical University, Kaohsiung, Taiwan; ^5^ Division of Colorectal Surgery, Department of Surgery, Kaohsiung Medical University Hospital, Kaohsiung Medical University, Kaohsiung, Taiwan; ^6^ Department of Surgery, Faculty of Medicine, College of Medicine, Kaohsiung Medical University, Kaohsiung, Taiwan; ^7^ Graduate Institute of Clinical Medicine, College of Medicine, Kaohsiung Medical University, Kaohsiung, Taiwan; ^8^ Division of Trauma and Surgical Critical Care, Department of Surgery, Kaohsiung Medical University Hospital, Kaohsiung Medical University, Kaohsiung, Taiwan; ^9^ Department of Emergency Medicine, Faculty of Post-Baccalaureate Medicine, College of Medicine, Kaohsiung Medical University, Kaohsiung, Taiwan; ^10^ Graduate Institute of Injury Prevention and Control, College of Public Health, Taipei Medical University, Taipei, Taiwan; ^11^ Center for Cancer Research, Kaohsiung Medical University, Kaohsiung, Taiwan; ^12^ Pingtung Hospital, Ministry of Health and Welfare, Pingtung, Taiwan

**Keywords:** ncRNAs, colorectal cancer, biomarkers, radiosensitivity, radioresistance

## Abstract

Colorectal cancer (CRC) is a global public health concern because of its high prevalence and mortality. Although radiotherapy is a key method for treating CRC, radioresistance is an obstacle to radiotherapy use. The molecular mechanisms underlying the radioresistance of CRC remain unclear. Increasing evidence has revealed the multiple regulatory functions of non-coding RNAs (ncRNAs) in numerous malignancies, including CRC. Several ncRNAs have been reported to be involved in the determination of radiosensitivity of CRC cells, and some have excellent potential to be prognostic biomarkers or therapeutic targets in CRC treatment. The present review discusses the biological functions and underlying mechanisms of ncRNAs (primarily lncRNA, miRNA, and circRNA) in the regulation of the radiosensitivity of CRC. We also evaluate studies that examined ncRNAs as biomarkers of response to radiation and as therapeutic targets for enhancing radiosensitivity.

## Introduction

Colorectal cancer (CRC) has a high incidence and mortality rate. Changes in dietary habits and lifestyle contribute to the increasing incidence of CRC (1). According to various statistics, CRC is the third leading malignancy and second most common cause of cancer-related deaths worldwide (2, 3). Treatment failure is the principal problem in the management of CRC. Treatment methods for rectal cancer are clinically different from those for colon cancer because of the anatomy and high risk of local recurrence associated with rectal cancer. Patients with rectal cancer account for approximately 40% of patients with CRC, and almost half of all patients with rectal cancer are diagnosed with locally advanced rectal cancer (LARC; stages II and III) (4). Neoadjuvant chemoradiotherapy (CRT) followed by radical resection is the standard of care for patients with LARC because this strategy provides improved locoregional control, disease-free survival (DFS), and sphincter-preserving rates (5, 6). For colon cancer, upfront radical resection is the principal therapy, and adjuvant chemotherapy is dependent on pathological features. However, several studies have indicated that neoadjuvant CRT can result in tumor downstaging and even pathological complete response (pCR), thereby facilitating complete tumor resection for locally advanced colon cancer (7, 8).

Because radiotherapy (RT) is one of the primary treatment approaches for CRC, the efficacy of RT can influence the eradication of cancer cells. However, radioresistance is an obstacle to the treatment of CRC. The targeting of nuclear DNA is the major mechanism that causes radiation injury in irradiated cells. Through the direct or indirect interactions between ionizing radiation (IR) and DNA, IR induces various DNA lesions that cause cellular damage and have the potential to kill cells. IR can interact directly with DNA molecules to damage DNA structures or interact indirectly with water molecules to produce reactive oxygen species (ROS) that then destroy DNA (9). Unpaired double-strand breaks (DSBs) are the most lethal form of DNA damage induced by IR (10). Molecules that can increase the number of DSBs or inhibit DNA repair pathways usually improve radiosensitivity (11). DSBs can lead to a series of DNA damage responses (DDRs), which are involved in the detection of DNA lesions, activation of signal transduction, and promotion of DNA repair; they also play a key role in cellular responses to IR (12). DDRs are triggered by the recognition of DNA damage by ataxia-telangiectasia mutated (ATM) and ataxia-telangiectasia- and RAD3-related (ATR) proteins, which are key mediators of DDRs (13). ATM activates the phosphorylation of p53, which then stabilizes itself and increases its accumulation in a nucleus (14). The activation of p53 results in the arrest of the cell cycle and the subsequent promotion of DNA repair or induction of apoptosis through specific pathways. Cell death or recovery is dependent on the level of DNA damage and p53 alterations (15). Two principal methods are involved in the repair of DSBs, namely homologous recombination (HR) and non-homologous end joining (NHEJ) (16). Radiation-induced DSBs principally activate the intrinsic apoptotic pathway. However, the extrinsic apoptotic pathway is also triggered by specific radiation doses (17). These radiation-associated regulatory pathways can be modulated by non-coding RNAs (ncRNAs), which are key mediators in the regulation of radiosensitivity.

**Figure 1:**
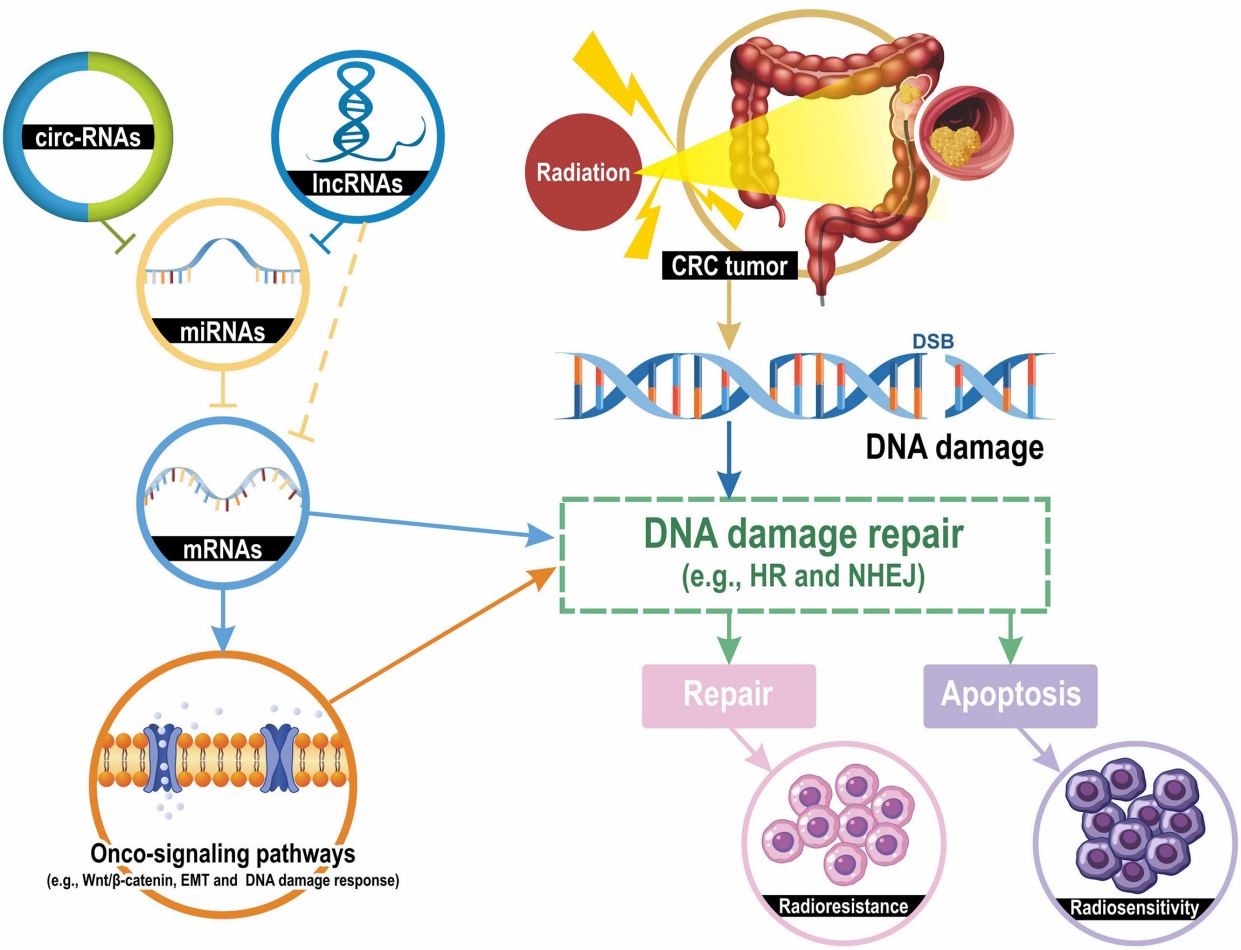
Mechanisms of ncRNAs contributing to CRC cell radiosensitivity. Radiation induces several types of DNA damage, and double-strand breaks (DSBs) are lethal. DNA damage activates the DNA damage repair pathway and the homologous recombination (HR) and the non-homologous end joining (NHEJ) are the most classic mechanisms for DSB repair. DNA damage repair results in two pathways: apoptosis and DNA repair, which lead to radiosensitivity or radioresistance. LncRNAs and circRNAs downregulate the expression levels of miRNA-targeted mRNAs by sponging their targeted miRNAs. mRNAs can directly regulate the DNA damage repair and indirectly modulate repair mechanisms through several onco-signaling pathways, such as Wnt/β-catenin, epithelial-mesenchymal transition (EMT), and DNA damage response. Taken together, the lncRNA-miRNA-mRNA and circRNA-miRNA-mRNA interaction networks modulate the radiosensitivity of CRC cells by regulating DNA damage repair pathways.

The human genome project indicates that less than 2% of the human genome encodes proteins and that more than 90% of the human genome is transcribed into ncRNAs, which are transcripts that lack the potential to code proteins (18, 19). ncRNAs have numerous types, and can generally be classified into two major categories. Transfer RNAs (tRNAs), ribosomal RNAs (rRNAs), and small nucleolar RNAs (snoRNAs) are classified as housekeeping ncRNAs, whereas circular RNAs (circRNAs), long ncRNAs (lncRNAs), microRNAs (miRNAs), tRNA-derived small RNA (tRFs), and small interfering RNAs (siRNAs) are classified as regulatory ncRNAs (20). LncRNAs contain transcripts with more than 200 nucleotides; they can be transcribed and synthesized like mRNAs, but they do not code proteins (21). By contrast, lncRNAs can regulate biological function through interactions with RNA and proteins. miRNAs are small, single-stranded ncRNAs that are approximately 20 nucleotides in length. By targeting mRNAs or other ncRNAs (e.g., lncRNAs and ciriRNAs), miRNAs can execute translational repression and mediate various biological processes (22). Initially, circRNAs were regarded as a by-product of alternative splicing that did not have a biological function (23). However, increasing evidence is indicating that circRNAs can function as alternative splicing mediators, miRNAs sponges, and parental gene expression regulators (24). Taken together, ncRNAs have multiple biological functions, including gene silencing, mRNA processing, chromatin remodeling, and transcriptional and translational regulation. The most frequently studied ncRNAs that are associated with the regulation of radiosensitivity are lncRNAs, miRNAs, and circRNAs. **Figure 1** illustrates the mechanisms of ncRNAs regulating radiosensitivity.

## Methods

We conducted a search of the databases of PubMed, the Cochrane Central Register of Controlled Trials, and Cochrane Reviews. We did not impose language and regional restrictions and searched the following terms in the format as follows: “non-coding RNA” AND “colorectal cancer “ AND “radiosensitivity” AND “radioresistance” and “miR” AND “colorectal cancer” AND “radiosensitivity” AND”radioresistance.” After searching PubMed with the keywords, we identified 357 relevant journal articles. All relevant studies and bibliographies were thoroughly reviewed to identify additional eligible studies.

### LncRNAs and response to radiotherapy—cell line analysis

Long ncRNAs (lncRNA) are characterized as RNAs that have a transcript length of more than 200 nucleotides and function as regulators in various cellular pathways, including DNA damage repair, alteration of cell cycle progression, and induction of resistance to apoptosis. LncRNAs regulate cellular processes by modifying chromatin and transcription, scaffolding for transcriptional factors, modulating mRNA translation, and sponging miRNAs (25). An increasing number of studies have indicated the role of lncRNAs in cancer. Dysregulated lncRNAs have been reported to induce radioresistance in various cancer cells, including CRC cells (26, 27). However, the mechanisms and biological function in CRC have not yet been fully elucidated. Here, we reviewed the literature on the effects of lncRNAs on the regulation of radiosensitivity in CRC. **Table 1** displays some lncRNAs that regulate radiosensitivity in CRC, and the targets of the lncRNA and clinical significance.

**Table 1:** Several lncRNAs involved in radiosensitivity of CRC.

lncRNA	Target	Radiosensitivity	Cell lines	Expression in clinical samples	Clinical significance	References
TLCD2-1	miR-193a-5p	decrease	CCL244 and HCT116	downregulated in CRC tissues	upregulated TLCD2-1 had decreased OS and DFS	(28)
ANRIL	miR-181a-5p	decrease	SW480	N/A	N/A	(29)
NEAT1	miR−448	increase	HCT116	N/A	N/A	(30)
LINC00958	miR-422a/MAPK1	decrease	SW480 and HCT8	upregulated in CRC tissues	upregulated LINC00958 had advanced T-stage and decreased OS and DFS	(26)
EGOT	miR-211-5p/ErbB4	decrease	HR8348 and Colo320	upregulated in rectal cancer tissues	upregulated EGOT had decreased OS	(31)
TTN-AS1	miR-134-5p/PAK3	decrease	HT29	N/A	N/A	(32)
HOTAIR	miR-93/ATG12	decrease	SW480 and HCT116	upregulated in CRC tissues and plasma	N/A	(27)
lnc-RI	miR-4727-5p/LIG4	decrease	HCT116 and HT29	N/A	N/A	(33)
OIP5-AS1	miR-369-3p/DYRK1A	decrease	LoVo and SW480	N/A	downregulated OIP5-AS1 had decreased OS N/A	(34)
TINCR	miR-137/TCF4	decrease	SW620 and HTC116	upregulated in CRC tissues	N/A	(35)
LINCE00630	BEX1	decrease	HCT116, SW480, LoVo, SW1116, and LS174	upregulated in the radioresistant tissues	upregulated LINCE00630 had decreased OS and recurrence-free survival	(36)

CRC, colorectal cancer; DFS, disease-free survival; lncRNA, long non-coding RNA; OS, overall survival; T-stage; tumor stage; N/A, not available.

Yu et al. identified the presence of lnc-TLCD2-1 in radiation-tolerant CCL244 cells and radiation-sensitive HCT116 cells and the presence of correlated lncRNA with radiation resistance. An *in vitro* study demonstrated that overexpressed lnc-TLCD2-1 induced radioresistance through the regulation of YY1/NF-кB-P65 by directly targeting miR-193a-5p in CRC cells, thus influencing the immune microenvironment of CRC (28). Sun et al. reported that the lncRNA antisense noncoding RNA in the INK4 locus (ANRIL) was upregulated in CRC cells and that the overexpression of ANRIL suppressed radiosensitivity by targeting miR-181a-5p and reversing the functions of chitooligosaccharide, a well-known radiation sensitizer. However, no clinical correlation or *in vivo* study has validated the effects of ANRIL (29). Pyroptosis (i.e., an inflammatory cell death) is regulated by gasdermins, and it functions as a key mediator in IR-induced damage in normal tissues (37). Su et al. published a study that investigated the role of lncRNA nuclear paraspeckle assembly transcript 1 (NEAT1) in the radiosensitivity of CRC cells. They indicated that lncRNA NEAT1 modulated IR-induced pyroptosis in HCT116 cells through miR−448 inhibition by regulating the expression of gasdermin E, a pyroptosis execution protein (30). Liang et al. reported that lncRNA LINC00958 was upregulated in CRC tissues and cell lines. They demonstrated that LINC00958 inhibited apoptosis and sensitivity to IR *in vitro* and enhanced tumor growth *in vivo*. LINC00958 modulated the radiosensitivity of CRC cells by targeting miR-422a, which suppressed MAPK1 expression (26). Li et al. reported that lncRNA eosinophil granule ontogeny transcript (EGOT) was upregulated in rectal cancer tissues and cells. Moreover, EGOT knockdown promoted the radiosensitivity of rectal cancer cells *in vitro* and *in vivo*, and EGOT regulated the radiosensitivity of rectal cancer cells by targeting the miR-211-5p/ErbB4 axis (31). Zuo et al. discovered that TTN antisense RNA 1 (TTN-AS1) lncRNA was overexpressed in CRC cells after exposure to IR and that TTN-AS1 knockdown improved the sensitivity of CRC cells to IR and promoted apoptosis by increasing the expression levels of Bax/Bcl2. Their findings suggested that TTN-AS1 lncRNA induced radioresistance by increasing PAK3 expression through the sponging of miR-134-5p and that the p21 and AKT/GSK-3β/β-catenin pathways may be involved in the regulation of radiosensitivity (32). Liu et al. reported that lncRNA homeobox transcript antisense intergenic RNA (HOTAIR) expression was upregulated in CRC tissues and cells and HOTAIR was also highly expressed in the plasma samples from patients with CRC after their RT and CRC cells following IR. HOTAIR knockdown enhanced radiosensitivity *in vitro* and *in vivo* through the upregulation of miR-93 and downregulation of ATG12-mediated autophagy (27). Liu et al. reported that the expression of radiation-induced long noncoding RNAs (lnc-RI) was low in radiosensitive CRC cells and that silenced lnc-RI promoted apoptosis caused by cell cycle arrest and induction of DSBs. The experimental results suggested that lnc-RI induced radioresistance by regulating NHEJ repair efficiency through the mediation of the lnc-RI/miR-4727-5p/LIG4 axis (33). Zou et al. investigated the role of lncRNA OIP5 antisense RNA 1 (OIP5-AS1) in modulating the radioresistance of CRC cells and discovered that the expression levels of lncRNA OIP5-AS1 and DYRK1A were low in radioresistant CRC cell lines. They performed functional assays, which verified that lncRNA OIP5-AS1 promoted radiation-induced apoptosis and radiosensitized CRC cells by regulating DYRK1A expression through the sponging of miR-369-3p (34). According to the starBase database (http://starbase.sysu.edu.cn/), LncRNA terminal differentiation-induced noncoding RNA (TINCR) was highly expressed in CRC tissues. Functional assays have verified that the knockdown of TINCR enhanced the radiosensitivity of radioresistant CRC cells and inhibited their stemness by inhibiting TCF4 through the regulation of miR-137 (35). Liu et al. demonstrated that INC00630 was highly expressed in CRC tissues and cells; their functional assays verified that high LINC00630 suppressed BEX1 expression and that the knockdown of LINC00630 promoted the radiosensitivity of CRC cells. Therefore, LINC00630 regulated the radioresistance of CRC cells partially by epigenetically inhibiting BEX1 (36).

### LncRNAs and response to radiotherapy—clinical findings

The clinical significance of several lncRNAs has been reported; therefore, they may serve as prognostic markers or therapeutic targets. Lnc-TLCD2-1 was reported to confer radioresistance on CRC cells. Furthermore, the expression levels of lnc-TLCD2-1 were lower in CRC tissues than in normal colon tissues, and patients with CRC who had a high expression of lnc-TLCD2-1 exhibited unfavorable DFS and overall survival (OS) (28). An *in vitro* study demonstrated that LINC00958 promoted cell proliferation and inhibited apoptosis and sensitivity to radiation. The associations between LINC00958 expression levels and clinicopathological features were analyzed in 63 patients with CRC, and the results revealed that patients with CRC with overexpressed LINC00958 had advanced T-stage, poorly differentiated tumor, and decreased DFS and OS (26). Lin et al. reported that lncRNA EGOT knockdown significantly radiosensitized rectal cancer cells *in vivo* and *in vitro*. Among the 40 analyzed patients with rectal cancer, those with upregulated EGOT had poor OS (31). Liu et al. explored the correlations between the levels of LINC00630 in tumor tissues and oncologic outcomes and reported a higher level of LINC00630 expression in the radioresistant cohort than in the radiosensitive cohort. Furthermore, high levels of LINC00630 were significantly associated with shorter OS and recurrence-free survival (36).

### MiRNAs and response to radiotherapy—cell line analysis

Radiation is usually combined with chemotherapy preoperatively or postoperatively to reduce locoregional recurrence in CRC. Tumor response to radiation varies considerably, and it involves numerous regulatory pathways (38). miRNAs are one of the key mediators of the radiosensitivity of CRC cells. **Table 2** lists several miRNAs that have been shown to be involved in the regulation of radiosensitivity and radioresistance in CRC. Several miRNAs have been identified as biomarkers of radioresistance or radiosensitization. Among radioresistance-related miRNAs, upregulated miR-106b induced radioresistance *in vitro* and *in vivo* through the suppression of *PTEN/PI3K/AKT* pathways and p21 in CRC cells (44). p21 was recognized as a tumor suppressor gene, and it functions as a mediator that can protect cancer cells from DNA damage (53, 54). Wu et al. reported that miR-96-5p induced the radioresistance of rectal cancer cells by inhibiting the *GPC3* gene and abnormally triggering the canonical Wnt signaling pathway (43). Liang et al. discovered that the expression of miR-32-5p was upregulated in CRC tissues and that a high level of miR-32-5p was associated with poor oncological outcomes. The suppression of miR-32-5p and the overexpression of *TOB1* gene increased the radiosensitivity of CRC cells, and *TOB1* was verified to be a direct target gene of miR-32-5p (42).

**Table 2:** Selected miRNAs involved in radiosensitivity of CRC cells.

miRNA	Target	Radiosensitivity	Cell lines	Expression in clinical samples/clinical significance	Animal study	References
Let‐7e	IGF1R	increase	HCT116	N/A	N/A	(39)
miR-1	N/A	increase	CCL244	downregulated in CRC tissues	N/A	(40)
miR-31	STK40	increase	LoVo and HCT116	upregulated in CRC tissues	N/A	(41)
miR-32-5p	TOB1	decrease	SW480	upregulated in CRC tissues/upregulated miR-32-5p had decreased OS and DFS	N/A	(42)
miR-96-5p	GPC3	decrease	HRC-99, HR-8348, SW1463, SW837, and RCM-1	N/A	Patient-derived xenograft	(43)
miR-106b	PTEN and p21	decrease	SW480 and SW620	N/A	SW480 and SW620 xenograft (8Gy)	(44)
miR-124	PRRX1	increase	SW480 and LOVO	decreased in blood of CRC patients after irradiation	SW480 xenograft (10Gy)	(45)
miR-145	SNAI1	increase	DLD1 and HCT116	N/A	patient-derived xenografts	(46)
miR-148a	c-Met	increase	HT29 and HCT116	upregulated in pCR patients/upregulated miR-148a had improved OS and DFS	HT29 xenograft (15Gy/3fx)	(47)
miR-185	IGF1R and IGF2	increase	radioresistant HCT116	N/A	N/A	(48)
miR-195	CARM1	increase	HCT-116 and HT-29	N/A	HT-29 xenograft; (8Gy)	(49)
miR-214	ATG12	increase	HCT116, SW480, Ls174.T and HT29	N/A	SW480 xenograft (10Gy)	(50)
miR-423-5p	Bcl-xL; apoptosis	increase	HCT116 and RKO	downregulated in radioresistant tissues	N/A	(51)
miR-1587	LIG4	increase	HCT116 and HT29	downregulated in CRC tissue	N/A	(52)

CRC, colorectal cancer; pCR, pathological complete response; N/A, not available.

Several miRNAs are correlated with radiosensitization. Zheng et al. reported that miR-195 sensitized CRC cells (HCT116 and HT29) to radiation by suppressing *CARM1 in vitro* and *in vivo* (49). Additionally, they found that the radiosentitizing effects of miR-195 on CRC cells could be reversed by restoring *CARM1* expression. The introduction of miR-145 can overcome *SNAI1*-mediated radioresistance in CRC cells; therefore, miR-145 can be used as a therapeutic agent for targeting cancer stem cells (46). Zhang et al. performed *in vitro* and *in vivo* studies and discovered that miR-124 could enhance the radiosensitivity of CRC cells by directly targeting *PRRX1*, which functioned as an epithelial–mesenchymal transition inducer and stemness mediator (45). miR-185 overexpression suppressed *IGF-1R* and *IGF2* and consequently improved the sensitivity of radioresistant CRC cells to IR (48). *IGF‐1R* has been associated with cellular resistance to radiation (55, 56). Samadi et al. demonstrated that let‐7e enhanced radiation‐induced apoptosis by directly inhibiting the expression of *IGF‐1R* in the HCT116 CRC cell line (39). Hu et al. indicated that miR-214 enhanced the radiosensitivity of CRC *in vitro* and *in vivo* by inhibiting radiation-induced autophagy and that miR overcame radioresistance by targeting *ATG12*, which functions as an autophagy-related gene (50). A study reported a higher expression of miR-31 in CRC tissues than in adjacent normal tissues; it also indicated that upregulated miR-31 levels increased the radiosensitivity of CRC cells by inhibiting *STK40*, which could inhibit the NF-κB signaling pathway (41). Downregulated miR-423-5p levels were identified in acquired radioresistant CRC cells and tissue specimens from patients with radiotherapy-resistant rectal cancer. The overexpression of miR-423-5p sensitized the acquired radioresistant CRC cells by targeting Bcl-xL, which is an antiapoptotic protein (51). Wu et al. discovered that miR-1 was downregulated in CRC tissues and cell lines and that the overexpression of miR-1 enhanced the sensitivity of CRC cells to IR and promoted apoptosis following irradiation (40). Liu et al. discovered that the expression of miR-1587 was lower in CRC tissues and cell lines than in normal colon tissues and cells, and they indicated that miR-1587 overexpression increased the formation of DSBs and radiosensitivity of CRC cells by directly targeting *LIG4* (52). Our previous study demonstrated that upregulated miR-148a enhanced radiosensitivity and promoted apoptosis in CRC cells by targeting *c-Met in vitro* and *in vivo* (47). A high level of miR-148a was correlated with improved tumor response to neoadjuvant CRT and survival outcomes.

### MiRNAs and response to radiotherapy—clinical findings

Because results of the role of miRNAs in the regulation of radiosensitivity in CRC cells have been promising, researchers have evaluated selected miRNAs in patients with CRC to predict RT responses. Differentially expressed miRNA profiling in cancer tissue samples can help identify potential biomarkers of response to RT. miRNA expression profiles were analyzed in tumor tissues collected from 80 rectal cancer patients from a randomized Swedish Rectal Cancer Trial. The decreased expression of miR-302a, miR-105, and miR-888 in the cancer tissues collected from RT and non-RT groups may regulate tumor response to RT in patients with rectal cancer (57). Machackova et al. evaluated tumor biopsy specimens collected from patients with LARC who received neoadjuvant CRT (20 responders and 20 non-responders) and reported that miR-487a-3p expression was significantly higher in the non-responder group than in the responder group (58). Hotchi et al. examined the tumor regression grade in LARC tissue samples and verified that miR-233 and miR-142-3p were associated with treatment response to CRT (59). Kheirelseid et al. reported that miR-16, miR-590-5p, and miR-153 were predictive biomarkers of pCR and that miR-519c-3p and miR-561 were significantly associated with a favorable response to CRT (60).

Although numerous studies have investigated tissue miRNAs to predict response to CRT, their results are highly inconsistent. The methods used to measure response to CRT include comparisons of pCR versus non-pCR or of responders versus non-responders through radiological or pathological downstaging, and this could have contributed to the inconsistencies in reported findings. Another possible explanation is that the studies did not recruit sufficient numbers of patients. Until now, only miR-21 has been identified as a biomarker for predicting pCR (61–63).

Studies have indicated that the stability of circulating miRNAs is excellent; thus, they can perform favorably as minimally invasive biomarkers. The circulating miRNAs obtained from liquid biopsies are promising and accessible predictive markers of response to RT. Guidance from circulating miRNAs can help clinicians in designing tailored therapies. Circulating miRNAs are difficult to measure because of their low concentrations in serum and plasma. However, advances in next-generation sequencing technology have led to the development of techniques for detecting small amounts of miRNAs in blood.

D’Angelo et al. analyzed 38 LARC patients and discovered that miR-125b was highly expressed in both the tissues and serum obtained from non-responders to CRT. They performed a receiver-operating-characteristic curve analysis by examining circulating miR-125b levels and serum carcinoembryonic antigen (CEA) levels and demonstrated that circulating miR-125b performed more favorably than serum CEA in the discrimination of treatment response (64). Hiyoshi et al. collected serum miRNAs from 94 patients with LARC who received neoadjuvant CRT, and they reported a lower expression of serum miR-143 in non-responders than in responders to CRT (65). Yu et al. reported that serum miR-345 expression was significantly lower in responders than in non-responders to CRT in both tissue and serum samples obtained from patients with LARC. In addition, low miR-345 levels were reported to be correlated with improved 3-year local recurrence-free survival (66). Joe et al. analyzed circulating miRNAs from patients with LARC and reported that none of the selected plasma miRNAs was associated with response to CRT. Furthermore, they revealed that the expression levels of circulating microRNAs changed during neoadjuvant CRT; moreover, the expression levels of plasma miRNAs were not always positively correlated with the level of miRNA expression in tumor tissues (67). Similar to the results for cancer tissue miRNAs, the results for circulating miRNAs are considerably inconsistent. Further studies involving the recruitment of large numbers of patients and the application of advanced biotechnology may help reduce this inconsistency.

### CircRNAs and response to radiotherapy—cell line analysis

circRNAs are highly stable and exhibit conservative behavior relative to linear RNAs. Increasing evidence has indicated that circRNAs can modulate gene expression at the transcriptional level by regulating the translation or stability of target RNAs through competition with miRNAs (68, 69). circRNAs regulate numerous biological processes, including proliferation, apoptosis, migration, and invasion. Several circRNAs have been discovered to posttranscriptionally modulate gene expression by sponging miRNAs. In the present study, we reviewed studies on the role of circRNAs in regulating the radiosensitivity of CRC. **Table 3** summarizes several circRNAs that modulate radiation response in CRC.

**Table 3:** Several circRNAs involved in radiosensitivity of CRC.

circRNA	Target	Radiosensitivity	Cell lines	Expression in clinical samples	Clinical significance	References
circ_0001313	miR-338-3p	decrease	SW480 and SW620	upregulated in CRC tissues	N/A	(70)
circ_0055625	miR-338-3p/MSI1	decrease	SW480 and SW620	upregulated in CRC tissues	upregulated circ_0055625 had decreased OS	(71)
circ_0067835	miR-296-5p/IGF1R	decrease	SW620 and HCT116	upregulated in CRC tissues and blood	N/A	(72)
circ-MFN2	miR-574-3p/IGF1R	decrease	LOVO, HCT-116, SW620 and SW480	upregulated in CRC tissues	upregulated circ-MFN2 had advanced TNM stage and decreased OS	(73)
circ_IFT80	miR-296-5p/MSI1	decrease	SW480 and SW620	upregulated in CRC patient serum	N/A	(74)

CRC, colorectal cancer; TNM, tumor node metastasis; N/A, not available.

**Figure 2:**
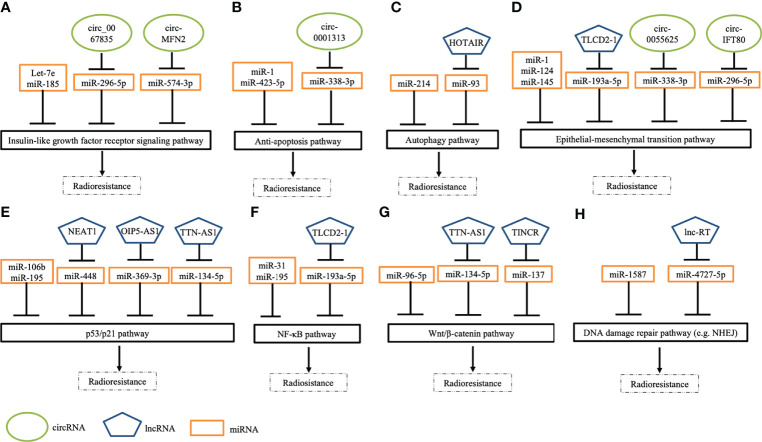
Schematic depicting several pathways of ncRNAs in modulating radiation response to CRC cells. **(A)** Several ncRNAs regulate radiosensitivity by targeting insulin-like growth factor receptor signaling pathways. **(B) **A few ncRNAs modulate cell apoptosis by regulating anti-apoptotic pathways. **(C)** Some ncRNAs modulate response to irradiation by mediating autophagy pathways. **(D)** Many ncRNAs manipulate epithelial-mesenchymal transition (EMT) by enhancing EMT (e.g., TLCD2-1) or inhibiting EMT (e.g., miR-124). **(E) **Numerous ncRNAs regulate DNA damage response pathways (e.g., p53 pathways) to modulate radiosensitivity. **(F)** Specific ncRNAs control NF-κB signaling pathways to overcome radioresistance. **(G)** Some ncRNAs mediate cellular response to irradiation *via* targeting Wnt/β-catenin. **(H)** Several ncRNAs regulate radiosensitivity through mediating DNA damage repair pathways, such as non-homologous end joining (NHEJ).

Wang et al. identified exosomal circ_0067835 to be upregulated in the serum obtained from patients with CRC who received RT. Circ_0067835 knockdown promoted radiosensitivity *in vitro* and *in vivo* by mediating the miR-296-5p/IGF1R axis (72). Liu et al. reported that circ-MFN2 expression was upregulated in CRC tissues and cells and that the knockdown of circ-MFN2 radiosensitized CRC cells *in vivo* and *in vivo*. In addition, circ-MFN2 knockdown inhibited cellular proliferation, invasion, and migration. Functionally, circ-MFN2 promoted proliferation and induced the radioresistance of CRC by upregulating miR-574-3p and downregulating IGF1R (73). Exosomal circ_IFT80 was reported to be upregulated in the serum and colon cancer cells of patients with CRC, and the overexpression of circ_IFT80 reduced the radiosensitivity of CRC cells through the inhibition of apoptosis. Exosomal circ_IFT80 could facilitate tumorigenesis and induce radioresistance by regulating the miR-296-5p/MSI1 axis. Furthermore, circ_IFT80 promoted tumor growth *in vivo* (74). Gao et al. reported that circ_0055625 and musashi homolog 1 (MSI1) were upregulated in CRC tissues and colon cancer cells. Circ_0055625 repression inhibited cell proliferation and promoted radiosensitivity *in vitro*, and it also suppressed tumor growth and enhanced radiation effects on inhibiting tumor growth *in vivo*. Circ_0055625 knockdown restrained the progression and radioresistance of CRC cells by downregulating MSI1 through the sponging of miR-338-3p (71). Wang et al. examined CRC tissues and cells and discovered an increase in the expression of circ_0001313 and a decrease in the expression of miR-338-3p. Functionally, circ_0001313 knockdown promoted the radiosensitivity of CRC cells by upregulating miR-338-3p. Moreover, circ_0001313 knockdown increased the caspase-3 activity in CRC cells under irradiation (70). **Figure 2** summarizes the relevant signaling pathways of ncRNAs contributing to CRC cell radiosensitivity.

### CircRNAs and response to radiotherapy—clinical findings

Some of the aforementioned circRNAs have clinical significance. Highly expressed circ-MFN2 induced proliferation, metastasis, and radioresistance in CRC cells. In a clinical study, the circ-MFN2 expression levels of 50 patients with CRC were examined, and upregulated circ-MFN2 was revealed to be significantly associated with advanced TNM stage, lymph node metastases, and unfavorable OS (73). Gao et al. discovered that circ_0055625 knockdown inhibited tumorigenesis and enhanced radiosensitivity in CRC cells; they examined 57 patients with CRC and discovered that high circ_0055625 and MSI1 expression levels were associated with a decreased survival rate among such patients (71). The regulatory networks of circRNA-miRNA-mRNA warrant further studies because their role in the regulation of radiosensitivity in CRC remains unclear. **Table 4** summarizes the clinical implications of the abovementioned ncRNAs.

**Table 4:** Several ncRNAs with clinical implications in CRC patients.

	No. of patients	Expression levels	5-year OS (%)	P value	5-year DFS (%)	P value	References
TLCD2-1	177	Low	68	0.016	80	0.008	(28)
		High	40		55		
LINC00958	63	Low	80	0.004	70	< 0.001	(26)
		High	48		24		
EGOT	40	Low	50	< 0.01	N/A	–	(31)
		High	26		N/A		
OIP5-AS1	N/A	Low	24	< 0.001	N/A	–	(34)
		High	40		N/A		
LINCE00630	50	Low	70	0.023	76	0.006	(36)
		High	45		40		
miR-32-5p	54	Low	60	< 0.05	38	< 0.05	(42)
		High	40		24		
miR-148a	51	Low	75	0.046	64	0.001	(47)
		High	95		100		
circ_0055625	57	Low	77	< 0.001	N/A	–	(71)
		High	24		N/A		
circ-MFN2	50	Low	75	0.003	N/A	–	(73)
		High	25		N/A		

CRC, colorectal cancer; DFS, disease-free survival; ncRNA, non-coding RNA; OS, overall survival; N/A, not available.

## Conclusion and future prospects

NcRNAs (primarily lncRNA, miRNA, and circRNA) constitute a complex regulatory network that responds to radiation injury. Through interactions with other ncRNAs, mRNAs, DNA, or proteins, ncRNAs can modulate cellular processes. Our knowledge of ncRNAs is still limited with respect to the variation-related molecular mechanisms for regulating radiosensitivity in patients with CRC. An increasing number of ncRNAs are being classified as oncogenes or tumor suppressor genes, which may modulate the sensitivity of cancer cells to irradiation. Therefore, understanding the underlying mechanism of ncRNAs in tumorigenesis and their regulatory pathways is a key aspect of cancer therapy development. Although ncRNAs have great potential as prognostic biomarkers for guiding precision medicine or as therapeutic targets for improving radiosensitivity in CRC treatments, the use of ncRNAs in clinical practice should be further studied.

With advances in biotechnologies, functional studies of ncRNAs can offer several novel perspectives on cancer treatment. Some ncRNAs have been proved to be associated with tumor stage, treatment efficacy, and prognosis. Several clinical trials have been initiated or are ongoing to investigate the role of ncRNAs in personalized cancer medicine, such as miR-31 (ClinicalTrials.gov Identifier: NCT03362684) and lncRNA CCAT1 (ClinicalTrials.gov Identifer: NCT04269746) in CRC, miR-21 in breast cancer (ClinicalTrials.gov Identifier: NCT05151224), and lncRNA HOTAIR in thyroid cancer (ClinicalTrials.gov Identifer: NCT03469544). The results of these clinical trials are worth looking forward to; moreover, further basic and clinical studies are warranted to investigate the effects of ncRNAs on the regulation of radiosensitivity and to test the clinical applications of ncRNAs in radiotherapy.

## Author Contributions

C-MH designed the study and wrote the draft. H-LT, Y-CC, and C-WH collected the related paper. C-CL and W-CS collected the tables and designed them. T-KC, Y-SY, and P-JC assisted in editing. J-YW and M-YH critically reviewed and revised the paper. All authors contributed to the article and approved the submitted version.

## Funding

This work was supported by grants through funding from the Ministry of Science and Technology (109-2314-B-037-046-MY3, MOST110-2314-B-037-097, MOST 111-2314-B-037-070-MY3, MOST 111-2314-B-037-049) and the Ministry of Health and Welfare (MOHW111-TDU-B-221-114014) and funded by the health and welfare surcharge of on tobacco products, and the Kaohsiung Medical University Hospital (KMUH110-0R37, KMUH110-0R38, KMUH110-0M34, KMUH110-0M35, KMUH110-0M36, KMUHSA11013, KMUH-DK(C)110010, KMUH-DK(B)110004-3) and KMU Center for Cancer Research (KMU-TC109A04-1) and KMU Office for Industry-Academic Collaboration (S109036), Kaohsiung Medical University. In addition, this study was supported by the Grant of Taiwan Precision Medicine Initiative and Taiwan Biobank, Academia Sinica, Taiwan, R.O.C.

## Conflict of Interest

The authors declare that the research was conducted in the absence of any commercial or financial relationships that could be construed as a potential conflict of interest.

## Publisher’s Note

All claims expressed in this article are solely those of the authors and do not necessarily represent those of their affiliated organizations, or those of the publisher, the editors and the reviewers. Any product that may be evaluated in this article, or claim that may be made by its manufacturer, is not guaranteed or endorsed by the publisher.
